# Gene expression profiles that shape high and low oil content sesames

**DOI:** 10.1186/s12863-019-0747-7

**Published:** 2019-05-16

**Authors:** Linhai Wang, Yanxin Zhang, Donghua Li, Komivi Dossa, Ming Li Wang, Rong Zhou, Jingyin Yu, Xiurong Zhang

**Affiliations:** 1 Oil Crops Research Institute of the Chinese Academy of Agricultural Sciences, Key Laboratory of Biology and Genetic Improvement of Oil Crops of the Ministry of Agriculture, Wuhan, 430062 China; 2 Centre d’Etudes Régional pour l’Amélioration de l’Adaptation à la Sécheresse (CERAAS), Route de Khombole, BP 3320, Thiès, Sénégal; 3 Laboratoire Campus de Biotechnologies Végétales, Département de Biologie Végétale, Faculté des Sciences et Techniques, Université Cheikh Anta Diop, BP 5005, Dakar-Fann, Code postal 107000 Dakar Sénégal; 4 USDA-ARS, Plant Genetic Resources Conservation Unit, 1109 Experiment Street, Griffin, GA 30223 USA

**Keywords:** *Sesamum indicum*, Seed, Carpel, Oil content, Gene, Expression profiling

## Abstract

**Background:**

Sesame (*Sesamum indicum*) can accumulate over 60% oil in its seed. However, low oil content genotypes with an oil content of less than 50% are also observed. To gain insights into how genes shape this variation, we examined 22 seed and carpel transcriptomes from 3 varieties of sesame with high and low oil content.

**Results:**

A total of 34.6~52.2% of the sesame genes were expressed with a RPKM greater than 5 in the 22 tissue samples. The expressed gene numbers tended to decrease in the seed but fluctuated in the carpels from 10 to 30 days post-anthesis (DPA). Compared with that of the low oil content sesames, the high oil content sesame exhibited more positive gene expression during seed development. Typically, genes involved in lipid biosynthesis were enriched and could distinguish the high and low genotypes at 30 DPA, suggesting the pivotal role of seed oil biosynthesis in the later stages. Key homologous lipid genes that function in TAG biosynthesis, including those that encoded glycerol-3-phosphate acyltransferase (*GPAT*), acyl-CoA:diacylglycerol acyltransferase (*DGAT*), and phospholipid:diacylglycerol acyltransferase (*PDAT*), were strengthened asynchronously at different stages, but the lipid transfer protein (*LTP*)-encoding genes, including *SIN_1019175, SIN_1019172* and *SIN_1010009*, usually were highlighted in the high oil content sesames. Furthermore, a list of 23 candidate genes was identified and predicted to be beneficial for higher oil content accumulation. Despite the different gene expression patterns between the seeds and carpels, the two tissues showed a cooperative relationship during seed development, and biological processes, such as transport, catabolic process and small molecule metabolic process, changed synchronously.

**Conclusions:**

The study elucidated the different expression profiles in high and low oil content sesames and revealed key stages and a list of candidate genes that shaped oil content variation. These findings will accelerate dissection of the genetic mechanism of sesame oil biosynthesis.

**Electronic supplementary material:**

The online version of this article (10.1186/s12863-019-0747-7) contains supplementary material, which is available to authorized users.

## Background

Sesame is an important and ancient oil crop. It has been cultivated for 5000 years in Asia and traditionally has been considered a high quality oil crop [1]. Analysis of the chemical components has shown that sesame seeds contain 48~62% oil by weight, and approximately 80% of the oil belongs to monounsaturated and polyunsaturated fatty acids, which are healthy for humans [2]. The high oil yield and quality earned sesame a label of “queen of oilseeds” [3]. With increasing knowledge of the dietary and health benefits of sesame, the consumption of sesame or sesame oil has increased significantly in Asia, Europe and America in recent years according to statistical data from the FAO.

With population growth, the global consumption of plant oils has increased by > 50% over the past decade [4]. Plant oil consumption is expected to double by 2040 with the increasing human population [5]. The steady demand for more edible plant oils has urged scientists to explore the genetic basis of oil biosynthesis and a high oil yield in crops. Sesame is ranked in the first class for oil content among the edible oil crops, such as soybean, rapeseed, peanut and olive. This feature, combined with its diploid character and small genome size of 357 Mb, make it an ideal plant model for studying oil biosynthesis or other traits [6].

Despite its high oil content, less is known about the genetic mechanisms or genes related to oil biosynthesis in sesame, because this topic has received very little attention from scientists. Indeed, the sesame has been considered an orphan crop for a long time. To date, a few genes, including *LTP*, *PDAT*, *SiPPO*, and *SiNST*, have been identified and predicted to be related to regulation of oil content in the sesame [7, 8]. Therefore, the genes involved in regulation of the sesame oil content and how they function during seed development still need to be uncovered. In contrast, extensive research into the genes and pathways involved in TAG or lipid biosynthesis has been performed in other crops, especially in the model plant *Arabidopsis thaliana* [9, 10]. The *A. thaliana* acyl-lipid metabolic reactions were reported to require at least 120 enzymatic reactions, and more than 700 genes encode the proteins and regulatory factors involved in these reactions [9, 11–15]. Therefore, a list of unanswered questions remains concerning which genes are involved and how they shape the high and low oil content in sesames [9].

Here, we report a comprehensive analysis of stage-specific sesame transcriptome profiles during the early (10 DPA, days post-anthesis), mid (20 DPA), mid-late (25 DPA), and later (30 DPA) seed development stages of a high oil content sesame (HO) and the two low oil content sesames (LOA and LOB). Because FA and TAG biosynthesis is tightly linked to photosynthesis and carbohydrate metabolism, which provide the carbon source for FA synthesis [16], the carpels corresponding to each seed sample at the different stages were also investigated. This study will facilitate the identification of differentially expressed genes and assessment of the gene expression patterns in developing seeds and carpels, which in turn will help identify some candidate genes that function in greater oil accumulation in the sesame.

## Results

### The sesame seed and carpel showed different gene expression patterns

**Fig. 1:**
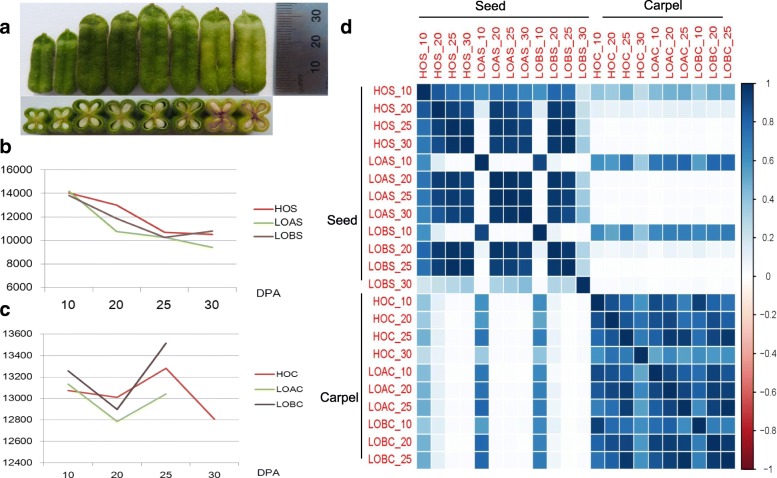
The development characteristics of the seed and carpel. **a** The carpels and seeds at 10, 20, 25 and 30 DPA. **b** The expressed gene numbers in the high and low oil content sesame seeds during development. **c** The expressed gene numbers in the high and low oil content sesame carpels during development. **d** The gene expression levels distinguished the seed and carpel after 20 DPA. HOS: Seed of high oil content sesame ZZM4728; LOAS: Seed of low oil content sesame ZZM3495; LOBS: Seed of low oil content sesame ZZM2161; HOS: Carpel of high oil content sesame ZZM4728; LOAS: Carpel of low oil content sesame ZZM3495; LOBS: Carpel of low oil content sesame ZZM2161

In the 22 seed and carpel samples (Fig. 1a, Additional file 1: Tables S1 and S2), 71.2% of the 27,148 sesame genes were expressed with a RPKM greater than 1. To guarantee the reliability of the gene expression profiles, genes with RPKM values less than 5 in at least one sample were filtered out. Following this standard, 9402~14,172 of the genes expressed in the samples were retained for further study (Fig. 1b, Additional file 1: Table S3), accounting for 34.6~52.2% of the total sesame genes. The expression levels of these genes ranged from 0.01 to 216,096.3 and averaged 60.2 RPKM, with the seed genes expressed at slightly higher levels than those of the carpels on average. The distinguished expression patterns between the sesame seeds and carpels were further highlighted using the expressed gene numbers and their corelationships (Fig. 1d, Additional file 2: Figure S1). From 10 to 30 DPA, the expressed gene numbers of the high and low oil content sesame seeds tended to decrease except for LOB, which showed a slight increase from 25 to 30 DPA. In the carpels, the numbers of expressed genes in the three varieties fluctuated from 10 to 25 DPA (Fig. 1c, Additional file 2: Figure S1). However, the gene expression profiles were not clear after 25 DPA, because the carpels of both LOA and LOB were senescent at 30 DPA.

### The later development stage is important for the high oil content

**Fig. 2:**
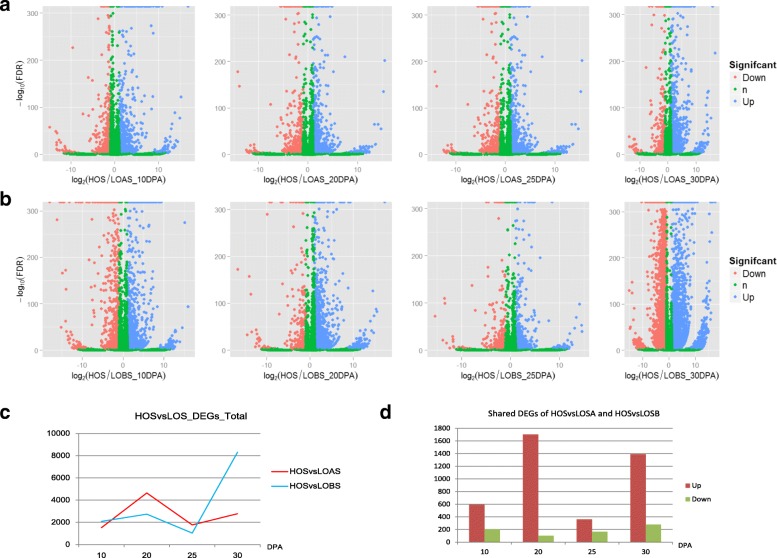
The differentially expressed genes in the high and low oil content sesames during seed development. **a** Volcano plot illustrating the DEGs between HO and LOA at different stages. The blue, red and green spots indicate the up-, down-, and normally regulated genes, respectively. **b** Volcano plot illustrating the DEGs between HO and LOB at different stages. **c** Numbers of the DEGs between the high and low oil content sesames during seed development. **d** The shared DEGs between HO and LOAS/LOBS

By scrutinizing differences in the expression profiles of the genes between the one high and the two low oil content sesame varieties, we found that 794, 1807, 528 and 1667 of the shared DEGs between HO and LOA/LOB were detected in the seeds at 10, 20, 25 and 30 DPA, respectively (Fig. 2). When the DEGs at different stages between HO and LOA/LOB were mapped to KEGG terms, most of the enriched pathways were shared by the seed and carpel from 10 to 25 DPA. However, the biological processes related to lipid biosynthesis, including unsaturated fatty acids, fatty acid metabolism and glycosphingolipid biosynthesis, were exclusively detected in the seed at 10 or 20 DPA, whereas few similar function processes were found in the carpels. Typically, lipid biosynthesis was strengthened at 30 DPA, with 8 of the 11 enriched pathways involving fatty acid biosynthesis, fatty acid elongation, fatty acid metabolism, biosynthesis of unsaturated fatty acids, sphingolipid metabolism, glycolysis/gluconeogenesis, galactose metabolism, and citrate cycle (TCA cycle) (Additional file 1: Table S4), indicating the pivotal role of the 30 DPA stage for lipid biosynthesis and accumulation.

### The homologous lipid genes involved in shaping the high oil content

Because many genes involved in lipid biosynthesis have been discovered from the model plant *A. thaliana* and other oil crops [9, 14, 16, 17], first we investigated the expression of the known lipid-related genes in the high and low oil content sesames. Referring to the *A. thaliana* lipid gene set, a list of 708 homologous lipid genes in sesame were identified [6]. A total of 569 of these genes were expressed in the three sesame varieties, of which 254 were differentially expressed genes (DEGs) for at least one time point between HO and LOA/LOB. In contrast, fewer homologous lipid genes were differentially expressed in the carpels, with only 79 shared DEGs detected (Additional file 1: Tables S5 and S6).

**Fig. 3:**
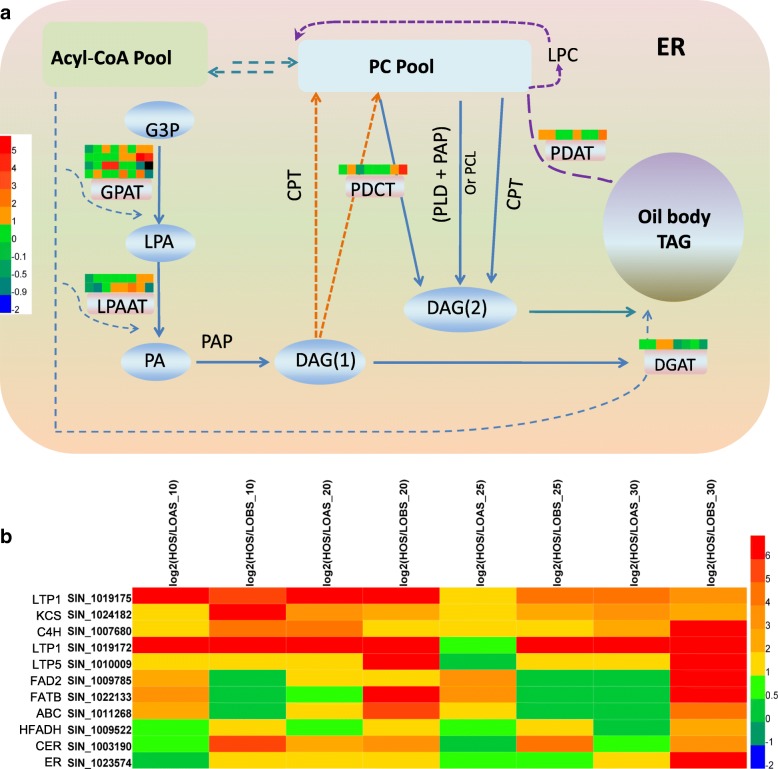
Expression patterns of the homologous lipid genes in the sesame seed. **a** Expression profiles of the known rate-limiting homologous lipid genes involved in sesame TAG biosynthesis. **b** The 11 genes homologous to *A. thaliana* lipid genes that were upregulated in the high oil content sesame seed

In *A. thaliana* and other plants, some genes have been revealed as rate-limiting steps in TAG biosynthesis, such as acetyl-CoA carboxylase (*ACCase*), acyl carrier protein (*ACP*), glycerol-3-phosphate acyltransferase (*GPAT*), phosphatidylcholine:diacylglycerol cholinephosphotransferase (*PDCT*), lysophosphatidic acid acyltransferase (*LPAAT*), acyl-CoA:diacylglycerol acyltransferase (*DGAT*), and phospholipid:diacylglycerol acyltransferase (*PDAT*) [9, 18]. These genes showed different expression patterns in sesame. *ACCase*, *ACP*, *PDAT*, *PDCT* and *LPAAT* were upregulated in the high oil content sesame seeds, but *GPAT* and *DGAT* were upregulated in both the seeds and carpels. The *PDAT* (*SIN_1010510*) expression trend was noted in the early and later development stages of the seed (10, 25 and 30 DPA), *DGAT* (*SIN_1027080*) was expressed in the middle stage (20 DPA), *GPAT* (*SIN_1009956*, *SIN_1012236*, *SIN_1021799* and *SIN_1026760*) was expressed in the middle and later stages, and *ACP* (*SIN_1010127* and *SIN_1016262*), *PDCT* (*SIN_1021891*) and *LPAAT* (*SIN_1006219* and *SIN_1011477*) were expressed in the later stages (25 and 30 DPA) and showed more striking expression patterns in the high oil content sesame (Fig. 3a). In addition to the above rate-limiting genes involved in TAG biosynthesis, we found 11 genes that were upregulated significantly (FDR ≤ 0.001) for at least two time points in the HO seed (Fig. 3b). Of them, 3 genes (*SIN_1019175*, *SIN_1019172* and *SIN_1010009*) belonged to the lipid transfer protein gene family (*LTP*) (Additional file 1: Table S7).

### Novel candidate genes that shaped sesame oil content variation

**Fig. 4:**
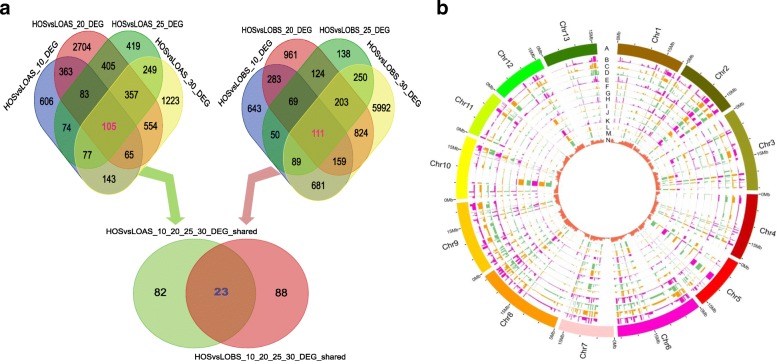
Shared and special DEGs between the high and low oil content sesames. **a** Venn diagrams of the shared and special DEGs in the seed at different stages. **b** Expression profiles of the 805 shared DEGs in the seed between the high and low oil content sesames. A: represents the 13 assembled pseudomolecules of the sesame; B, E, H and K correspond to HO at 10, 20, 25 and 30 DPA, respectively; C, F, I and L correspond to LOA; D, G, J and M correspond to LOB; N: represents the gene density (mRNA, 500-kb window)

Previous studies showed that the sesame had fewer lipid homologous genes (708) than *A. thaliana* (737) and the soybean (1298) [6]. However, these lipid homologous genes may not represent all of the genes involved in sesame lipid biosynthesis due to its higher oil content, and some novel function genes are expected to be discovered. Because genes with functions in the regulation of oil content variation usually expressed differently in different genotypes during seed development, we focused on DEGs between the high and low oil content sesames. The numbers of DEGs changed with seed development from 10 to 30 DPA (Figs. 2 and 4a). The high oil content sesame had more upregulated genes, especially at 20 and 30 DPA (Fig. 2c and d), indicating that HO was more positive in terms of oil biosynthesis.

In total, 805 DEGs were identified between the high and low oil content sesames at two or more time points (Fig. 4b). These genes may be involved in improving the oil content. Of these genes, 45 were found to be homologous to *A. thaliana* lipid genes (Additional file 1: Table S8), and 341 had tight corelationships with these homologous lipid genes (*R* ≥ 0.9). GO term enrichment indicated that these genes had the molecular functions oxidoreductase activity, iron ion binding, lipid transport, catalytic activity, monooxygenase activity, lipid binding, haem binding, and electron carrier activity, which indicated that they acted mainly in oxidation-reduction and lipid metabolic processes (Additional file 1: Table S9).

**Fig. 5:**
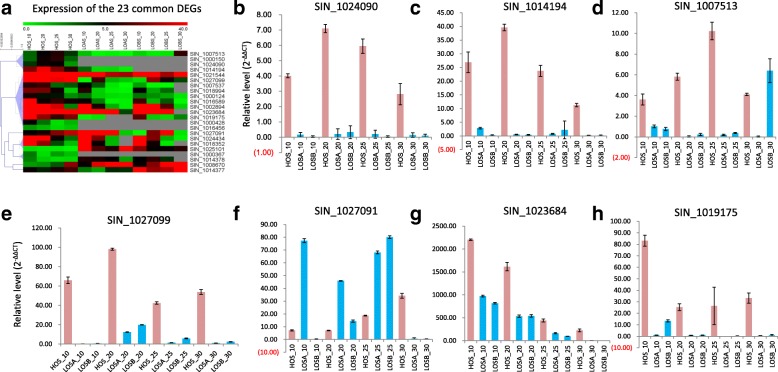
Expression patterns of the core candidate genes for high oil content. **a** Expression patterns of the 23 common DEGs in the seed between the high and low oil content sesames. **b**-**h** Quantitative RT–PCR validation of 7 candidate genes for high oil content, including SIN_1024090, SIN_1014194, SIN_1007513, SIN_1027099, SIN_1027091, SIN_1023684 and SIN_1019175. Pink bars represented the high oil content sesame, and blue bars represented the low oil content sesames

Notably, 23 DEGs were commonly detected during seed development (Figs. 4a and 5a, Additional file 1: Table S10). These genes were the core candidate genes for the high oil content or were responsible for oil biosynthesis. Of them, genes *SIN_1007513* and *SIN_1019175* were homologous to the lipid genes *AT2G30490.1* and *AT3G51590.1* of *A. thaliana,* respectively, and belonged to the cinnamate 4-hydroxylase and lipid transfer protein type 1 gene families. *SIN_1023684* was annotated as a plant lipid transfer protein and functioned in the biological process lipid transport (GO: 0006869). *SIN_1027091* encoded a lipase and was involved in lipid metabolic process (GO: 0006629). *SIN_1014194*, *SIN_1024090* and *SIN_1027099* were all upregulated in the high oil content sesame (Fig. 4c) and should be novel genes, because no public reference information was available for them. Quantitative RT-PCR confirmed the expression differences of the above seven genes (Fig. 5).

### Sesame seeds and carpels cooperated in oil accumulation

**Fig. 6:**
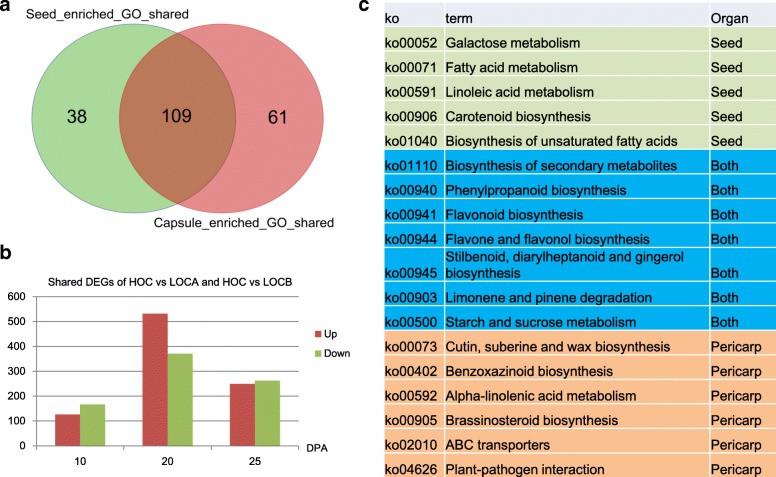
The relationships between the seed and carpel during development. **a** The shared and special GO terms of the expressed genes in the two tissues. **b** Numbers of the shared DEGs between HO and the two low oil content sesames in the carpels at different stages. **c** The shared and special pathways of the DEGs between the high and low oil content sesames in the two tissues

Because sesame seed is embedded in carpels, the relationship between the two tissues in carbohydrates and other nutrients transport is interesting. Across the different stages, it showed there 6994 and 10,565 genes were commonly expressed in the sesame seeds and carpels, respectively (Additional file 2: Figures S2 and S3), and 90.8% of the common genes in the seed were also included in the carpel set. The function analysis found that the two gene sets were enriched in 147 and 170 GO terms, respectively, with 109 of them shared by the two tissues (Fig. 6a). This finding indicates that the seed and carpel have similar biological processes or cooperate tightly during their development, especially in transport, catabolic process and small molecule metabolic process (Additional file 1: Table S11). Further evidence for cooperation between the seeds and carpel was observed in some gene networks. For example, *COPII* is a type of vesicle coat protein that functions to transport proteins from the rough endoplasmic reticulum to the Golgi apparatus [19, 20]. Although typically the genes for the *COPII* vesicle coat were enriched in the sesame carpels, the endoplasmic reticulum and Golgi apparatus were found to be shared by the two tissues.

Regarding the DEGs in the carpels between the high and low oil content sesames (Fig. 6b), many of the DEGs shared the biological processes of the seeds, such as biosynthesis of secondary metabolites, phenylpropanoid biosynthesis, and flavonoid biosynthesis (Fig. 6c). However, generally the reactions related to lipid biosynthesis, including galactose, fatty acid, and linoleic acid metabolism, were highlighted in the seed, and the ATP-binding cassette (*ABC*) transporters, cutin, suberine and wax biosynthesis, and plant-pathogen interaction were detected in the carpels. *ABC* transporters constitute a ubiquitous superfamily of integral membrane proteins that are responsible for translocation of a diverse assortment of substrates ranging from ions to macromolecules, including lipids, across membranes [21], which may improve the ability of HO to transport carbon to the seed for lipid biosynthesis. For the 55 DEGs that were common between the high and low oil content sesame carpels (Additional file 1: Table S12, in Additional file 2: Figure S4), five genes (*SIN_1021544*, *SIN_1000367*, *SIN_1016456*, *SIN_1000428* and *SIN_1014194*) were also included in the 23 common DEGs, as mentioned above. According to their homologs in *A. thaliana*, the first three genes were predicted to function in many tissues with multiple physiological and biochemical roles [22–24], although the functions of *SIN_1000428* and *SIN_1014194* are still unclear. To summarize, these results suggest that the seed and carpel cooperate tightly during oil biosynthesis.

## Discussion

### The key stages for increased oil accumulation

After pollination, the sesame ovary takes approximately 30 to 40 days to mature, and most cultivars exhibit a rapid increase in the accumulation of oil and other nutrients from 10 DPA that reaches a peak at approximately 30 DPA [25–27]. During the seed development stages, many metabolic reactions take place to enable the biosynthesis and accumulation of metabolites, which result in the final seed composition of approximately 55% oil, 20% protein and other components. The active gene numbers plus the DEGs between the high and low oil content sesames showed that 20 DPA was the vigorous development stage for both the seed and carpel, with more genes upregulated in the high oil content sesame. However, most of these genes were not related to lipid biosynthesis directly. In contrast, the pathways related to lipid biosynthesis were significantly enriched and regulated in HO at 30 DPA, especially those for FA biosynthesis. The FA chain length (up to 18 carbons) and the level of saturated FAs are determined by the FA synthesis pathway, which needs glycolysis with hexose and/or triose as the predominant carbohydrate to deliver carbon for FA synthesis by entering the plastid [17]. Thus, the former stages (before 25 DPA) prepare substrates for FA and TAG biosynthesis in the later stages. Thus, the gene expression patterns were consistent with oil accumulation, and 30 DPA was the key stage for increased oil biosynthesis.

### Sesame homologous lipid genes for high oil content

The de novo assembly of TAG from glycerol-3-phosphate (*Gly3P*) and acyl-CoAs begins with the export of free FAs from the plastid. The first acylation of *Gly3P* at the sn-1 position is catalysed by *GPAT* and *LPAAT* to form *LPA*, followed by *PAP*, and a third acylation is performed by *DGAT* [17, 28], which is known as the Kennedy pathway [29]. *ACCase*, *ACP*, *PDAT*, and *PDCT* are also involved in the process. These genes are very important and act as rate-limiting steps for TAG or lipid biosynthesis in other plants [9, 18]. For example, *ACCase* was reported to be the first rate-limiting enzyme in the de novo synthesis of fatty acid by catalysis of acetyl-CoA carboxylation to form malonyl-CoA [30]. The study showed that the *GPAT* and *DGAT* genes in the pathway were significantly differentially expressed between the high and low oil content sesames in both tissues (seed and carpel), whereas *ACCase*, *ACP*, *PDAT*, *PDCT* and *LPAAT* were only differentially expressed in the seed. These genes may represent the key lipid homologous genes that shape the high or low oil content in the sesame. However, because fewer studies on sesame lipid genes are available, the functions of these genes (except for *PDAT* and *DGAT*, which have been reported to be related to sesame oil content variation) still need further evidence.

Another important homologous lipid genes is that encoding *LTP*. *LTP* is known to interact with a variety of different lipids and to facilitate the movement of lipids between membranes by binding them [31]. Based on their molecular sizes and sequence similarity, the *LTP* genes are classified into types (i.e., *LTP1*, *LTP2*, *LTP3* and so on) [32]. In a previous study, expansion of the *LTP1* gene family was predicted to shape the characteristics of sesame with a high oil content, and a genome wide association study showed that some *LTP* genes regulated oil content variation [8]. Here, *SIN_1019175* and *SIN_1019172* were *LTP1*-encoding genes, and *SIN_1010009* belonged to *LTP5*. These genes are also believed to be beneficial for more oil accumulation through efficient lipid transport.

### The novel candidate genes related to oil biosynthesis in the sesame

For the dominant proportions of oil in the sesame seed, a great number of genes are believed to participate and regulate its transport and biosynthesis. A previous study showed that sesame had 708 lipid homologous genes, which was less than that of other oil crops, such as *Glycine max* (1298) [6], *Arachis hypogaea* (1500 unigenes) [33, 34], and *Brassica napus* (2229) [35], and was also less than that of the model plant *A. thaliana* (737). Among the 708 predicted homologous lipid genes in the sesame, 139 were expressed at a low level. The study determined that 805 genes might be related to the high and low oil content in the sesame, of which 341 were correlated to lipid homologous genes. In particular, 23 genes were associated with the high and low oil content variation based on their expression patterns, suggesting their potential roles in lipid accumulation in combination with their function annotations. These genes will be targets for a future genetic study and verification using other functional methods.

### Methods to increase the sesame oil content

Unlike the seeds of rapeseed and soybean, sesame seeds are free of chlorophyll from initiate to maturity like Fig. 1a displayed, thus its photosynthetic capacity is limited, and can barely fix carbon for tissue substance biosynthesis [36]. Thus, cooperation between the carpel and seed is necessary for oil biosynthesis and substance accumulation, which involves many processes [9, 37]. Here, we showed that 64.1 to 74.1% of the enriched GO terms that functioned through seed and carpel development were shared by the two tissues. The DEGs between the high and low oil content sesames as well as the enriched pathways were predominantly shared by the seed and carpel. These results suggest tight cooperation between the sesame seed and carpel for oil biosynthesis.

Plant breeders and metabolic engineers have tried to increase oil production in seeds for decades. The strategies have mainly relied on increasing the supply of upstream substrates (source control) and the ‘sink’ strength in the last steps of the TAG metabolic pathways [17]. The first strategy not only includes strengthening of substrate biosynthesis but also the flux or transport capability to the next step in the lipid metabolic pathway. The strategies have been successful applied in maize and soybean [38–40]. In the present study, generally metabolism using starch and sucrose as the substrates was strengthened in both the carpel and seed from 10 to 25 DPA. The LTP1 to LTP5 gene families and ABC transporters, which function to increase the transport capability of the substrate flux, were also observed to be strengthened in the seed or carpel of HO compared to that of the low oil content sesame. In future studies, genetic manipulation of these genes together with other key genes may be an efficient method to improve the sesame seed oil content.

## Conclusions

The study of 22 sesame seed and carpel transcriptomes revealed the gene expression profiles during sesame seed and carpel development from 10 to 30 DPA and found that the high oil content sesame was more active than the low oil content sesames with more upregulated genes. Additionally, the later development stage played a pivotal role in the increased oil accumulation. In addition to the known rating-limit lipid genes, 805 sesame homologous lipid genes plus other genes that were differentially expressed between the high and low oil content varieties were predicted to be involved in the regulation of sesame oil content variations. In particular, 23 of these genes were considered as the core candidate genes and warrant further validation. The study also illustrated tight cooperation between sesame seeds and carpels during seed development and oil biosynthesis according to the gene expression patterns and their functions. Collectively, the study has uncovered the key stages and the candidate genes that shape high and low oil content sesames, which can be used to breed genetically improved sesame varieties with a high oil content.

## Methods

### Materials

The three sesame varieties (ZZM4728, ZZM3495 and ZZM2161, numbered HO, LOA and LOB, respectively) (Additional file 1: Table S1) used in the study were provided by the National Sesame Medium-Term Genebank (Wuhan, China). The oil content of ZZM2161 and ZZM3495 is low (48.4 and 50.95% of their seed weight, respectively). ZZM4728 is a high oil content variety with a percentage of 59.1.

### Planting and sampling

The three varieties were planted under the same growth and experimental conditions. Flowers were tagged every five days post-anthesis six times. The capsules at 10, 20, 25 and 30 DPA were sampled from 10 plants for each variety (Fig. 1a), and then the seeds and carpels were separated on ice. The seeds or carpels from different plants were mixed equally and used to represent the samples at 10, 20, 25 and 30 DPA.

### RNA extraction and library preparation

The carpels of ZZM2161and ZZM3495 at 30 DPA were too old to be used for RNA extraction. Finally, 22 samples were obtained and subjected to RNA-seq analysis (Additional file 1: Table S1). HOS, LOAS and LOBS represented the seeds and HOC, LOAC and LOBC represented the carpels of the three varieties, respectively. RNA extraction and sequencing were performed according to the procedure described by Wang et al. [41]. Briefly, total RNA was extracted from these samples with the TRIzol reagent (Invitrogen Corp.), and the mRNA was purified from the total RNA using the Oligotex mRNA Midi Kit (Qiagen, Germany). The mRNA quantity and quality were evaluated with the ND-1000 NanoDrop spectrometer (NanoDrop Technologies, USA) and a 2% denatured agarose gel. Then, these RNAs were transcribed into double-stranded cDNAs using the SMART cDNA Library Construction kit (Clontech, USA) following the manufacturer’s protocol. Adapters were ligated to the targeted fragments, and suitable fragments (200 ± 25 bp) were selected for PCR amplification. The short fragments were used for library construction for RNA-seq.

### Data generation and quality assessment

The 22 cDNA libraries generated from the sesame seeds and carpels were subjected to paired-end sequencing using the Illumina Hiseq 2000 platform. The libraries were sequenced for paired-end reads of 90 bp. The base qualities of the RNA-seq reads were checked using the FastQC software (http://www.bioinformatics.babraham.ac.uk/projects/fastqc/) to determine the bases. Paired-end reads containing more than 5% ambiguous residues (Ns) and those containing more than 10% bases with a Phred quality score of less 20 were removed. After cleaning and quality assessment, the remaining reads were referred to as “clean reads” [37]. Finally, 25.6–27.3 million clean reads with a 90-bp length were obtained for each sample (Additional file 1: Table S2). By setting the parameter that no more than one mismatch was allowed in the alignment, 67.7–87.7% of the clean reads were uniquely mapped to the sesame reference genome using SOAPaligner/SOAP2 [6, 42], with 50.3–70.7% of the reads uniquely mapping to the predicted gene model regions.

### Statistical analysis of gene expression

The gene expression levels were calculated based on the read numbers that uniquely mapped to the sesame genome sequence [6] and were normalized to the number of Reads per Kilobase of transcript per Million mapped reads (RPKM) using the Cufflinks 2.0 software [43]. The differentially expressed genes (DEGs) were identified for some samples as described by Chen et al. [44] and Wang et al. [45]. The Poisson distribution [46] and false discovery rate (FDR) were used to determine the threshold *P*-value in multiple tests. Here, a FDR ≤ 0.001 and absolute value of log_2_Ratio ≥ 1 were used to determine the significance of the DEGs [47].

### Gene annotation and enrichment analysis

GO (Gene Ontology, http://www.geneontology.org) is an international standardized gene function classification system. KEGG (Kyoto Encyclopedia of Genes and Genomes, http://www.kegg.jp/) pathways represent knowledge of the molecular interaction and reaction networks. The target genes of the study were annotated with GO terms and metabolic pathways. The enrichment analyses were performed with R language packages according to Wang et al. [45]. The online tool GOSlimAuto was used to summarize the gene GO terms [48].

### Homologous genes involved in lipid biosynthesis

The 736 acyl-lipid genes of *A. thaliana* were downloaded from http://aralip.plantbiology.msu.edu. Employing blastp (E-value <1e-5, identity > 30%), their homologous models in the sesame were predicted using the Reciprocal Best Blast Hit (RBH) method [49, 50]. These genes were sorted according to different cellular functions and gene families.

### Real-time quantitative PCR (qRT-PCR)

The expression profiles of some genes were validated with RT-PCR referring to Wang et al. [45] with the LightCycler® 480II Real-Time PCR Detection System (Roche Diagnostics, Rotkreuz, Switzerland). Each sample was run in triplicate on the same plate with a negative control that lacked cDNA. The sesame *actin7* gene was used as a positive control. The relative expression levels of the target genes were calculated using the 2^-ΔΔCT^ method [51].

## Additional files

Additional file 1: **Table S1.** Materials and samples; **Table S2.** Overview of the generated data; **Table S3.** The total expressed genes with a RPKM greater than 5; **Table S4.** The enriched KEGG pathways of the DEGs between HO and LO in the seeds and carpels at different stages; **Table S5.** The 254 DEGs for more than one stage between HOS and LOAS/LOBS; **Table S6.** The 79 DEGs for more than one stage between HOC and LOAC/LOBC; **Table S7.** The 11 upregulated lipid homologous genes; **Table S8.** The 805 DEGS that appeared in two or more stages between the high and low oil content sesames; **Table S9.** The enriched GO terms for the 45 lipid homologous genes and 341 co-expressed genes; **Table S10.** Function annotation of the 23 core DEGs in the seed between the HO and LO sesames; **Table S11.** Shared and special enriched GO terms in the seeds and carpels; **Table S12.** Function annotation of the 55 core DEGs in the capsule between the HO and LO sesames. (XLSX 68 kb)

Additional file 2: Clustering of the 22 samples based on gene expression; **Figure S2.** Unique and shared expressed genes in the seeds of the high and low oil content sesames; **Figure S3.** Special and shared expressed genes in the carpels of the high and low oil content sesames; **Figure S4.** The shared DEGs between the high and low oil content sesames at different stages in the carpels. (DOCX 2298 kb)

## Abbreviations

ABC: ATP-binding cassette
ACCase: Acetyl-CoA carboxylase
ACP: Acyl carrier protein
DEG: Differentially expressed gene
DGAT: Acyl-CoA: Diacylglycerol acyltransferase
DPA: Days post anthesis
FA: Fatty acids
FDR: False discovery rate
GO: Gene ontology
GPAT: Glycerol-3-Phosphate Acyltransferase
KEGG: Kyoto encyclopedia of genes and genomes
LPAAT: Lysophosphatidic acid acyltransferase
LTP: Lipid transfer protein gene family
PDAT: Phospholipid diacylglycerol acyltransferase
PDCT: Phosphatidylcholine:diacylglycerol cholinephosphotransferase
RPKM: Reads per kilo base of exon model per million mapped reads
TAG: Triacylglycerol
