# Supplementary material for: Gene expression profiles that shape high and low oil content sesames
Source: BMC Genet. 2019 May 16;20:45. doi: 10.1186/s12863-019-0747-7 (PMC6521469; doi:10.1186/s12863-019-0747-7)
Supplement: Supplementary file 2 — Clustering of the 22 samples based on gene expression; Figure S2. Unique and shared expressed genes in the seeds of the high and low oil content sesames; Figure S3. Special and shared expressed genes in the carpels of the high and low oil content sesames; Figure S4. The shared DEGs between the high and low oil content sesames at different stages in the carpels. (DOCX 2298 kb) [file 12863_2019_747_MOESM2_ESM.docx]

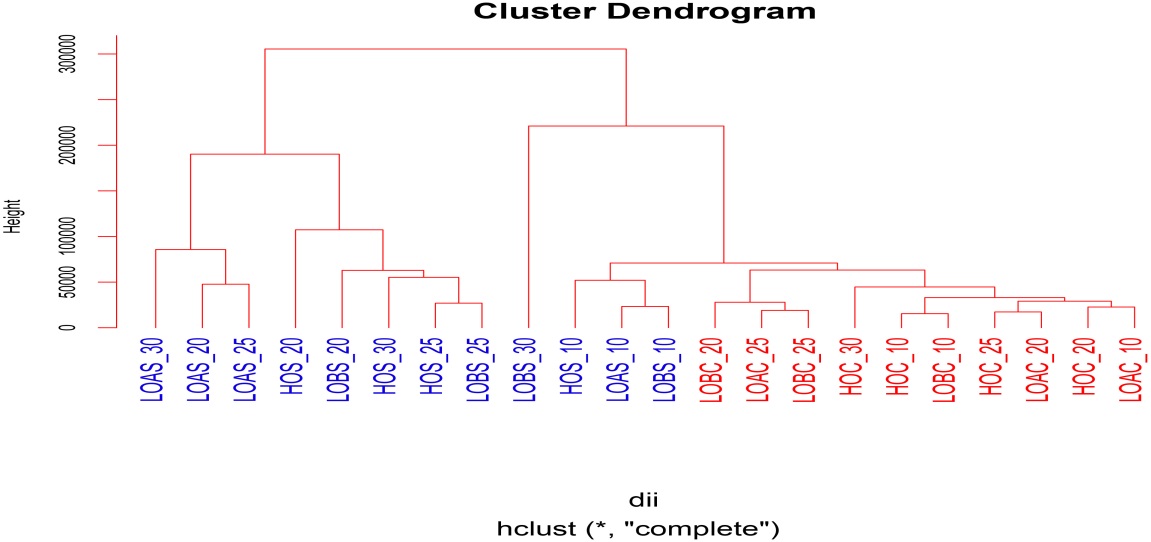


**Figure S1**. Clustering of the 22 samples based on gene expression. HOS: seed of the high oil content sesame variety ZZM4728, LOSA: seed of the low oil content sesame variety ZZM3495, LOSB: seed of the low oil content sesame variety ZZM2161; HOC: capsule of the high oil content sesame variety, LOCA: capsule of the high oil content sesame variety ZZM3495, LOCB: capsule of the low oil content sesame variety ZZM2161.


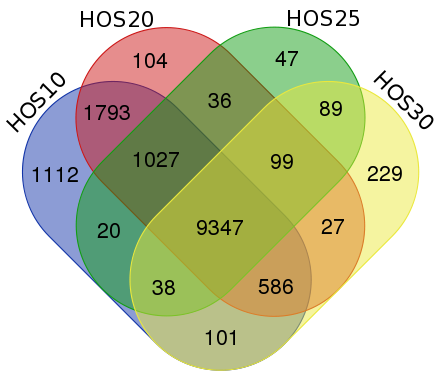

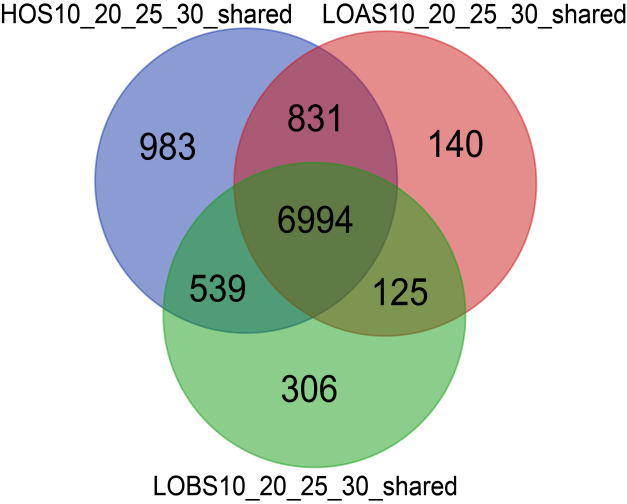

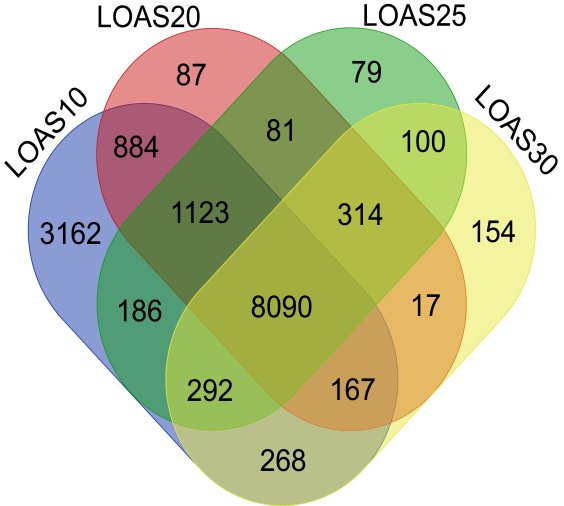

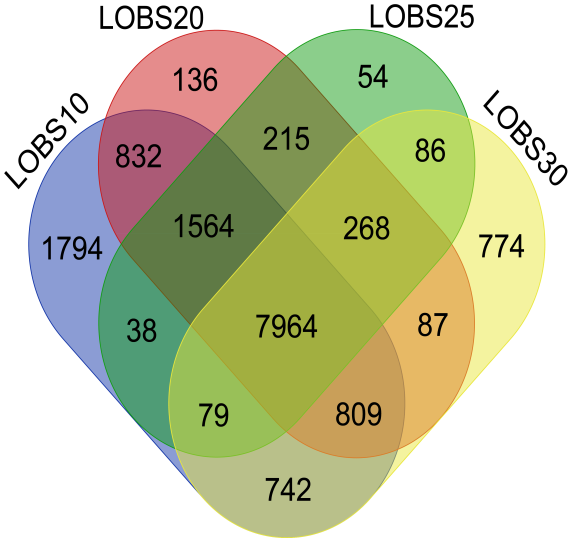


**Figure S2**. Unique and shared expressed genes in the seeds of the high and low oil content sesames.


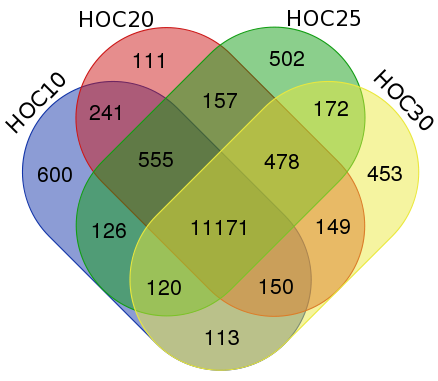

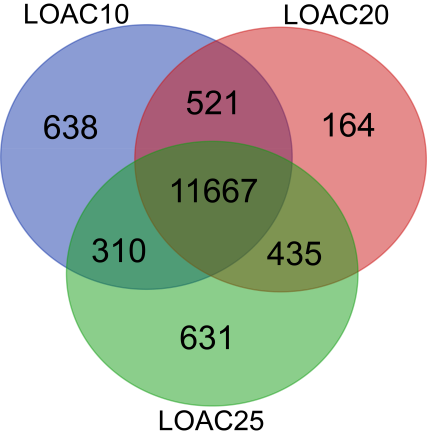

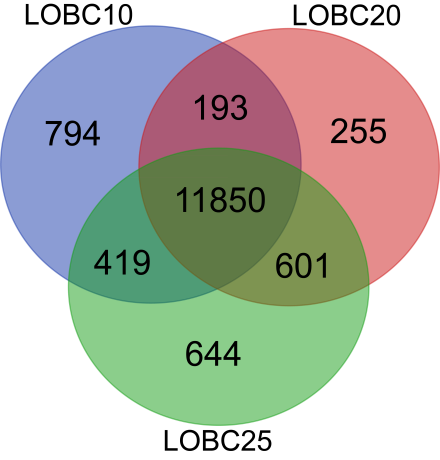

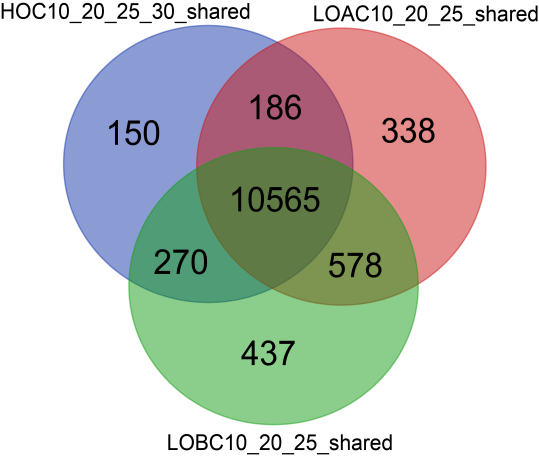


**Figure S3**. Special and shared expressed genes in the carpels of the high and low oil content sesames.


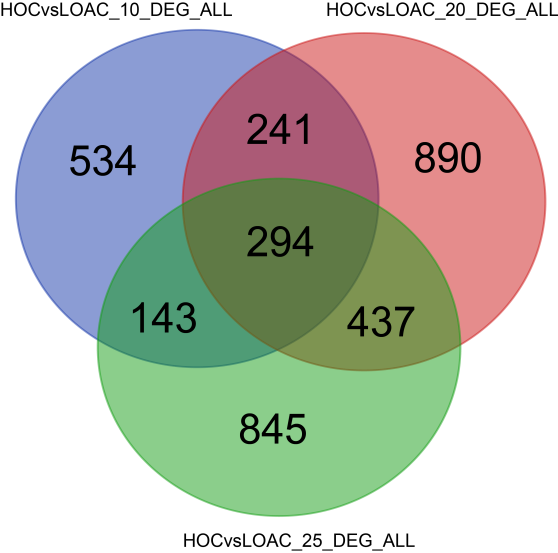


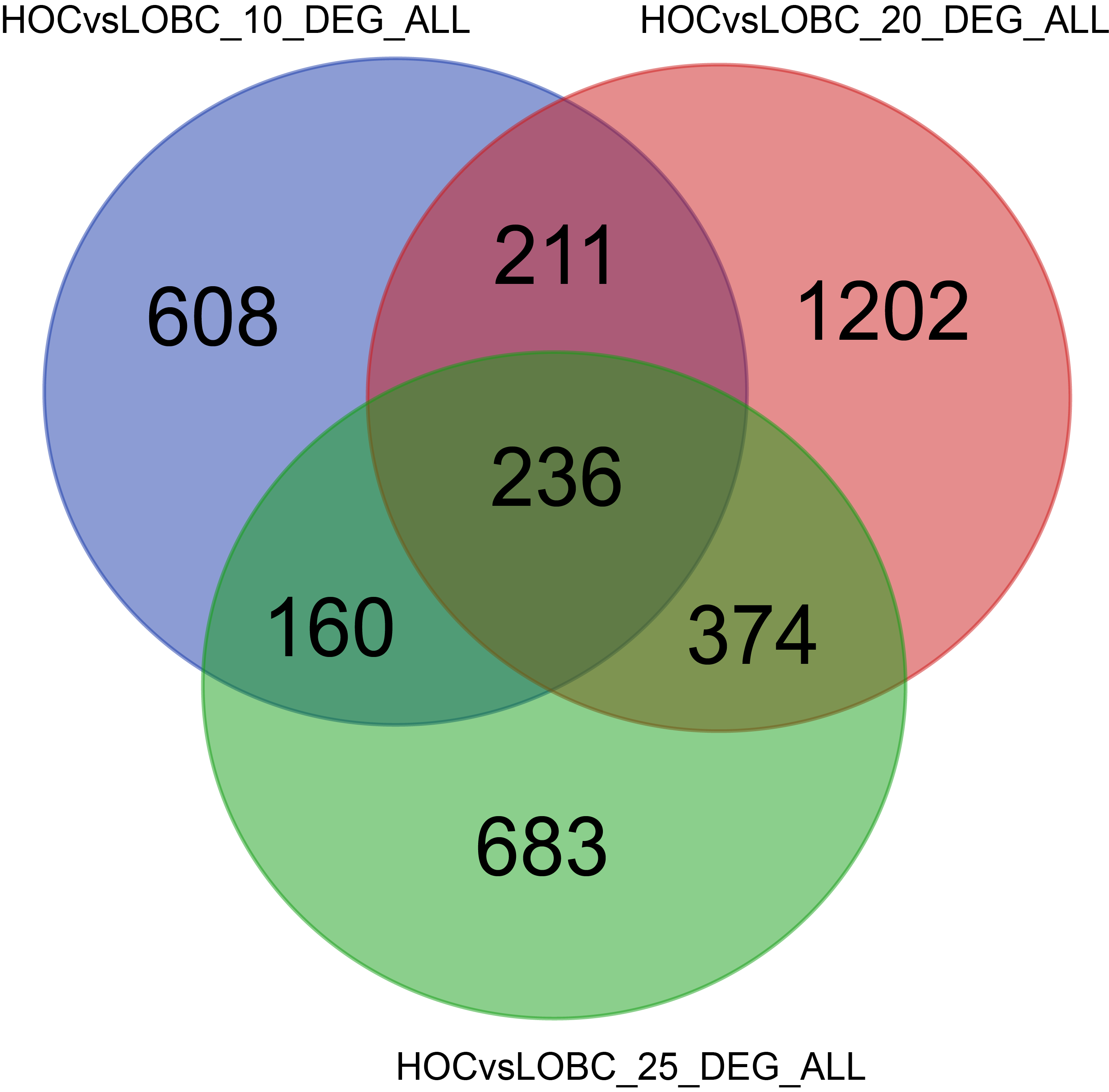

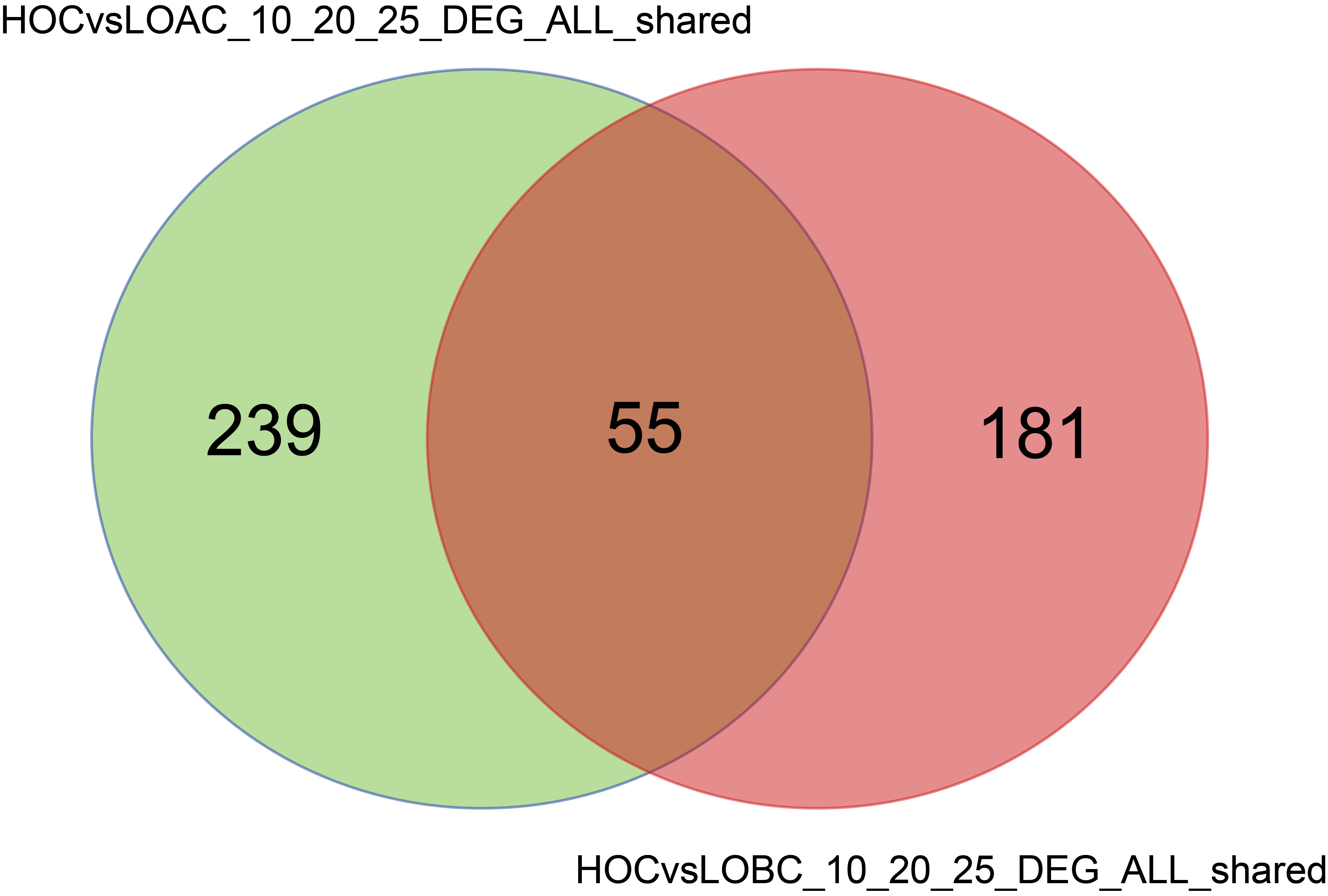


**Figure S4**. The shared DEGs between the high and low oil content sesames at different stages in carpels.
